# Association mapping via a class of haplotype-sharing statistics

**DOI:** 10.1186/1753-6561-1-s1-s123

**Published:** 2007-12-18

**Authors:** Andrew S Allen, Glen A Satten

**Affiliations:** 1 Department of Biostatistics and Bioinformatics, Duke University, Hock Plaza, Suite 1102, 2424 Erwin Road, Durham, North Carolina 27705, USA; 2 Duke Clinical Research Institute, Duke University, North Pavilion, 2400 Pratt Street, Durham, North Carolina 27705, USA; 3 Centers for Disease Control and Prevention, Mailstop K-23, 4770 Buford Highway, Atlanta, Georgia 30345, USA

## Abstract

We present a class of haplotype-sharing statistics useful for association mapping in case-parent trio data. The framework presented allows derivation of novel tests as well as new simplified variance estimators for previously proposed tests. We give an overview of this framework and apply four such tests to the simulated data of Genetic Analysis Workshop 15. We find that these haplotype-based statistics result in greater power and better risk locus localization than the single locus single-nucleotide polymorphism analysis.

## Background

Haplotype-sharing methods attempt to utilize insights from population genetics while maintaining the simplified statistical model used for association studies in genetic epidemiology. Coalescent models suggest that for some diseases, chromosomes of affected persons share a more recent common ancestor than a randomly selected pair of chromosomes. If a disease-causing mutation is relatively recent, haplotypes of affected persons may be identical by state (IBS) over a longer region near a risk locus than would be found among randomly selected haplotypes. Thus, haplotype sharing attempts association mapping by looking for regions where the patterns of similarity in IBS among haplotypes of affected persons differs from that found in random haplotypes.

In a recent paper, we derived the distribution of some previously proposed and novel haplotype-sharing tests [1]. Here, we give an overview of these results and apply them to the Genetic Analysis Workshop 15 (GAW15) Problem 3 data.

## Methods

For the *i*^th ^of *n*case-parent trios, let *H*_1*i *_and *H*_2*i *_be the paternal transmitted and untransmitted haplotypes, while *H*_3*i *_and *H*_4*i *_denote the maternal transmitted and untransmitted haplotypes. Assume haplotypes having *L *loci, so that there are 2^*L *^possible haplotypes. Let S_*k*_(*H*_1_, *H*_2_) measure the similarity between haplotypes *H*_1 _and *H*_2 _at a fixed locus *k*. Many similarity metrics are possible; here we measure similarity by the maximum information length contrast, the number of loci *H*_1 _and *H*_2 _share IBS looking upstream and downstream from a fixed locus *k*. Let S_*k *_be the matrix having (*i*, *j*)^th ^element S_*k*_(*H*_*i*_, *H*_*j*_). Let $\hat{\pi }$, $\hat{\rho }$, and $\hat{p}$ denote vectors of haplotype frequency estimators for untransmitted, transmitted, and all haplotypes respectively, obtained under phase uncertainty.

We consider statistics of the form

$$U_{k}\left(\gamma \right)=\gamma^{T}S_{k}\left(\hat{\rho }-\hat{\pi }\right).$$

It is possible to show that taking *γ *= $\hat{p}$ yields the numerator of the haplotype-sharing statistics considered by each of van der Meulen and te Meerman [2], Bourgain et al. [3], Tzeng et al. [4], and Zhang et al. [5], though these statistics differ in the computation of their variances. Writing these "standard" haplotype sharing tests in the form Eq. (1) allows us to interpret them as looking for differences between vectors $\hat{\rho }$ and $\hat{\pi }$ that are in the direction of $\hat{p}$^*T*^S_*k*_, i.e., in the direction of sharing with the parental haplotypes. The form of *U*_*k*_(*γ*) also allows us to derive a simple formula for its variance. We make explicit the fact that *γ *is often a function of the data by writing $\hat{\gamma }$. Using Slutsky's theorem [6, Section 1.5.4], as long as $\hat{\gamma }\overset{p}{\to }\gamma_{0}\neq 0$ under the null hypothesis, Var{*U*_*k*_($\hat{\gamma }$)} can be estimated by ${\hat{\gamma }}^{T}S_{k}\hat{\Sigma }S_{k}\hat{\gamma }$, where $\hat{\Sigma }$ is the empirical variance estimator of ($\hat{\rho }$ - $\hat{\pi }$). This variance estimator is considerably simpler than those previously proposed, and is valid even with phase uncertainty and for stratified populations [1]. Use of *γ *= $\hat{p}$yields the statistic $T_{\hat{p}}=U_{k}^{2}\left(\hat{p}\right)/\text{Var}\left\{U_{k}\left(\hat{p}\right)\right\}$, which we refer to as the *p *test. Another choice, *γ *= $\hat{\rho }$, was used by Levinson et al. [7], who contrasted sharing in transmitted haplotypes, ${\hat{\rho }}^{T}S_{k}\hat{\rho }$, with the cross product ${\hat{\rho }}^{T}S_{k}\hat{\pi }$ to give ${\hat{\rho }}^{T}S_{k}\hat{\rho }-{\hat{\rho }}^{T}S_{k}\hat{\pi }={\hat{\rho }}^{T}S_{k}\left(\hat{\rho }-\hat{\pi }\right)$. We call this the *rho *test.

An appealing choice of *γ *is ($\hat{\rho }$ - $\hat{\pi }$), as this direction weights differences in haplotypes by their differences in frequency (Gerard te Meerman, personal communication). However, Slutsky's theorem no longer applies as $\left(\hat{\rho }-\hat{\pi }\right)\overset{p}{\to }0$ under the null hypothesis. Instead, we use the fact that $U_{k}\left(\hat{\rho }-\hat{\pi }\right)={\left(\hat{\rho }-\hat{\pi }\right)}^{T}S_{k}\left(\hat{\rho }-\hat{\pi }\right)$ is a quadratic form whose distribution is a mixture of independent *χ*^2 ^variates, with weights given by the eigenvalues of the matrix $\hat{\Sigma }$S_*k*_. Following Imhof [8], we approximate this weighted *χ*^2 ^distribution using a three-moment approximation. We refer to the resulting test as the *cross *test.

Finally, we note that because the *p *test uses $\gamma =\hat{p}=\frac{1}{2}\left(\hat{\rho }+\hat{\pi }\right)$, while the *cross *test uses *γ *= ($\hat{\rho }$ - $\hat{\pi }$), the two tests appear to be looking at sharing in orthogonal directions; hence, a *combined *test seems desirable. Thus, we seek the distribution of $T_{\hat{p}}+U_{k}\left(\hat{\rho }-\hat{\pi }\right)={\left(\hat{\rho }-\hat{\pi }\right)}^{T}\left[\frac{{\hat{p}}^{T}S_{k}S_{k}\hat{p}}{{\hat{p}}^{T}S_{k}\hat{\Sigma }S_{k}\hat{p}}+S_{k}\right]\left(\hat{\rho }-\hat{\pi }\right)$. Once again, this is a quadratic form whose distribution is a mixture of independent *χ*^2 ^variates, with weights given by the eigenvalues of the matrix $\hat{\Sigma }\left[\frac{{\hat{p}}^{T}S_{k}S_{k}\hat{p}}{{\hat{p}}^{T}S_{k}\hat{\Sigma }S_{k}\hat{p}}+S_{k}\right]$, and we approximate this distribution as in Imhof [8].

### Application to GAW15 data

We compare the *rho*, *p*, *cross*, and *combined *tests by applying them to the GAW15 Problem 3 simulated "loose" SNP set for chromosome 6. We extracted 200 trios from each of 100 replicates by taking the first affected sibling and their parents from the first 200 families in each data set. We used only 200 trios both to speed up computation and because the effect of the risk locus on chromosome 6 was so strong that a reduced data set seemed more realistic. We used the answers to guide our analysis throughout. Specifically, we focused on a 10-cM region (45 cM to 55 cM) around the DR rheumatoid arthritis risk locus on chromosome 6 (DR locus is at 49.45557055 cM). In each data set we scanned the region using haplotype windows of 10 loci. The windows were shifted through the region two SNPs at a time so that if the first window started with SNP1 the next window would start with SNP3. The *rho*, *p*, *cross*, and *combined *tests were computed for each window and the transmission disequilibrium test (TDT) was applied to each SNP in the region. Estimates of haplotype frequencies required for the computation of the test statistics were computed using the software package HAPLORE [9]. In each data set we compute the max{-log_10_(*P*_*value*_)} for each test (where the max is taken over loci) and note this value and its position (for the haplotype-based tests the location is taken as the average location of SNPs 5 and 6 in the window), which we take as an estimate of the location of the risk locus. An average localization bias for each test was then computed by averaging the distance between the estimated locations and the true risk locus position over the 100 data sets. We compared the empirical distributions of -log_10_(*P*_*value*_) values for each test at three loci to investigate the effect of increasing distance from the true disease locus on the performance of each test.

## Results and discussion

**Figure 1:**
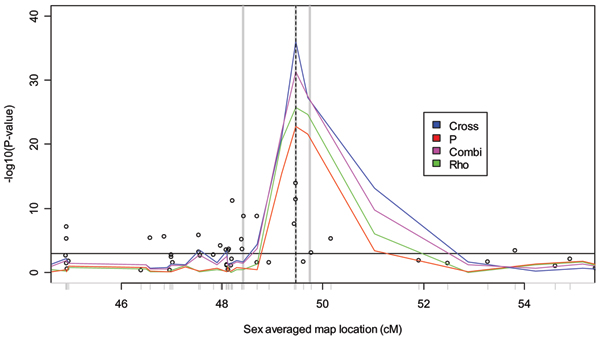
**Analysis of Replicate 1 in a 10-cM region containing risk locus**. Risk locus indicated by dotted vertical line. TDT results indicated by circles. SNP locations indicated by gray tick marks. Gray vertical lines represent loci further investigated in Figure 2. Horizontal black line indicates Bonferroni-corrected 0.05 significance level.

**Figure 2:**
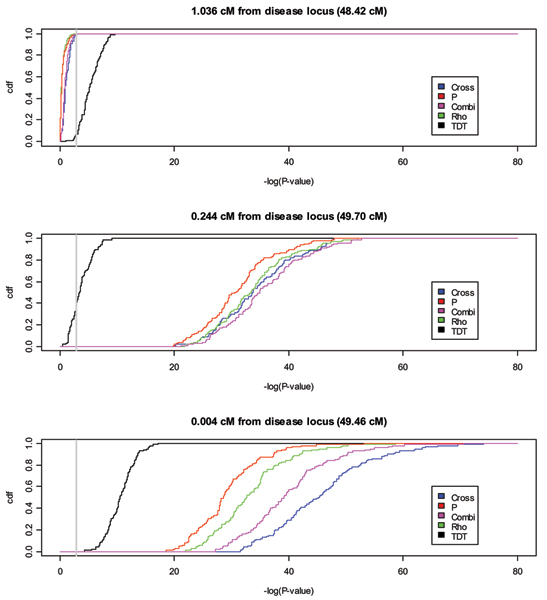
**Empirical distribution function of -log_10_(*P*_*value*_) values for three loci over 100 replicates**. Location of loci are indicated by gray vertical lines in Figure 1 and are shown in order of decreasing distance from the true disease locus. Gray vertical line indicates Bonferroni-corrected 0.05 significance level.

Figure 1 presents the results of the *rho*, *p*, *cross*, *combined*, and TDT tests in the 10-cM region of the chromosome 6 risk locus for Replicate 1. Three things are apparent from this analysis. First, the haplotype-based methods seem to be more powerful than the TDT, yielding much larger -log_10_(*P*_*value*_) values. Second, the haplotype-based methods seem to localize the risk locus well. Finally, the haplotype-based methods seem to be more concentrated around the risk locus, being both larger at the locus and dropping more quickly away from the risk locus than the TDT. Visual inspection of other data replicates suggests the same pattern; to confirm, we investigated each of the above points systematically. First, in order to summarize the power of the various tests we report the first quartile, median, mean, and third quartile of the max{-log_10_(*P*_*value*_)}of each test over the 100 replicates (Table 1). We see that the haplotype-based methods are consistently higher and that the *cross *test performs best among all tests. Next, we report the localization bias and MSE of the TDT and each of the haplotype sharing tests (Table 1). Here, once again, the *cross *test appears to do better than the others, though we note that the small biases involved make it difficult to make conclusions. Finally, Figure 2 presents the empirical distribution functions of -log_10_(*P*_*value*_) values for each test statistic at three different loci. Our findings are consistent with the observations in Replicate 1: the haplotype-based methods have larger -log_10_(*P*_*value*_) values at the risk locus and drop off more quickly away from the risk locus than the TDT throughout the replications. In particular, at 1.036 cM from the disease locus, essentially all replicates have a non-significant test statistic (i.e., values that fall to the left of the gray vertical line in Figure 2) for all of the haplotype sharing tests while most replicates have a significant TDT. By 0.244 cM the situation has changed, and all replicates have significant haplotype-sharing tests while about 40% of replicates have a non-significant TDT. At 0.004 cM from the disease locus, all tests are significant, but the superiority of the *cross *statistic for these data is more readily apparent.

**Table 1:** Bias and power summaries of 100 data replicates

	Bias		Power indicated by max{-log_10_(*P*_*value*_)}			
		
Test	Mean	MSE	1^st ^quartile	Mean	Median	3^rd ^quartile
*rho*	0.135	0.032	30.3	34.9	34.3	38.2
*p*	0.168	0.040	27.9	31.9	31.8	35.0
*cross*	0.015	0.002	39.2	45.7	45.3	50.3
*combined*	0.050	0.010	35.4	40.4	39.3	44.2
TDT	0.024	0.016	11.8	13.9	13.8	15.6

## Conclusion

We presented an overview of a new framework for deriving haplotype-sharing statistics and applied four such statistics to the GAW15 simulated data. Our findings suggest that these haplotype-based statistics can result in greater power and better risk locus localization compared to the single-SNP (TDT) analysis. The framework presented allows visualization of relationships between tests and computation of simplified estimators of the asymptotic distribution of the test statistics. This second feature is quite important because previous estimators have been complex or have depended on permutation procedures, making systematic power studies difficult or impossible.

## Competing interests

The author(s) declare that they have no competing interests.

## References

[B1] Allen AS, Satten GA (2007). Statistical models for haplotype sharing in case-parent trio data. Hum Hered.

[B2] Van der Meulen M, te Meerman G (1997). Haplotype sharing analysis in affected individuals from nuclear families with at least one affected offspring. Genet Epidemiol.

[B3] Bourgain C, Genin E, Quesneville H, Clerget-Darpoux F (2000). Search for multifactorial disease susceptibility genes in founder populations. Ann Hum Genet.

[B4] Tzeng J, Devlin B, Wasserman L, Roeder K (2003). On the identification of disease mutations by the analysis of haplotype similarity and goodness of fit. Am J Hum Genet.

[B5] Zhang S, Sha Q, Chen H, Dong J, Jiang R (2003). Transmission/disequilibrium test based on haplotype sharing for tightly linked markers. Am J Hum Genet.

[B6] Serfling R (1980). Approximation Theorems of Mathematical Statistics.

[B7] Levinson D, Kirby A, Slepner S, Nolte I, Spijker G, te Meerman G (2001). Simulation studies of detection of a complex disease in a partially isolated population. Am J Med Genet (Neuropsych Genet).

[B8] Imhof J (1961). Computing the distribution of quadratic forms in normal variables. Biometrika.

[B9] Zhang K, Sun F, Zhao H (2005). HAPLORE: a program for haplotype reconstruction in general pedigrees without recombination. Bioinformatics.

